# Analysis of the Effect of Tillage Depth on the Working Performance of Tractor-Moldboard Plow System under Various Field Environments

**DOI:** 10.3390/s22072750

**Published:** 2022-04-02

**Authors:** Yeon-Soo Kim, Sang-Dae Lee, Seung-Min Baek, Seung-Yun Baek, Hyeon-Ho Jeon, Jun-Ho Lee, Wan-Soo Kim, Jong-Yeal Shim, Yong-Joo Kim

**Affiliations:** 1 Smart Agricultural R&D Group, Korea Institute of Industrial Technology (KITECH), Gimje 54325, Korea; kimtech612@kitech.re.kr (Y.-S.K.); sdlee96@kitech.re.kr (S.-D.L.); 2 Department of Smart Agriculture Systems, Chungnam National University, Daejeon 34134, Korea; bsm1104@naver.com (S.-M.B.); kelpie0037@gmail.com (S.-Y.B.); jhh5888@naver.com (H.-H.J.); dlwnsgh211@naver.com (J.-H.L.); 3 Department of Bio-Industrial Machinery Engineering, Kyungpook National University, Daegu 41566, Korea; wansoo.kim@knu.ac.kr; 4 Agricultural Machinery Certification Team, Foundation of Agricultural Technology Commercialization and Transfer (FACT), Iksan 54667, Korea; tison21c@efact.or.kr; 5 Department of Biosystems Machinery Engineering, Chungnam National University, Daejeon 34134, Korea

**Keywords:** tillage depth, soil properties, moldboard plow, field load measurement system, agricultural tractor

## Abstract

The purpose of this study was to analyze the tillage depth effect on the tractor-moldboard plow systems in various soil environments and tillage depths using a field load measurement system. A field load measurement system can measure the engine load, draft force, travel speed, wheel axle load, and tillage depth in real-time. In addition, measurement tests of soil properties in the soil layer were preceded to analyze the effect of field environments. The presented results show that moldboard plow at the same tillage depth had a wide range of influences on the tractor’s working load and performance under various environments. As the draft force due to soil–tool interaction occurred in the range of 5.6–17.7 kN depending on the field environment, the overall mean engine torque and rear axle torque were up to 2.14 times and 1.67 times higher in hard and clayey soil, respectively, than in soft soil environments. In addition, the results showed tractive efficiency of 0.56–0.73 and were analyzed to have a lugging ability of 67.8% with a 44% maximum torque rise. The engine power requirement in hardpan was similar within 3.6–9.6%, but the power demand of the rear axle differed by up to 18.4%.

## 1. Introduction

Static or dynamic mechanical loads are physical stresses on a mechanical system or component. Some mechanical loads are utilized as design loads for mechanical systems [1]. To design agricultural machinery such as a tractor or determine an appropriate power source, it is important to predict the required power and load characteristics of each major power transmission unit [2]. Therefore, measuring mechanical loads in the laboratory or the field using appropriate test methods is essential for optimal design and performance evaluation. In particular, agricultural tractors operate in unstructured working environments, unlike commercial vehicles; thus, it is essential to secure load data in consideration of various agricultural working environments.

The working load of the agricultural tractor occurs according to the soil resistance caused by the soil–tool interaction during tillage operation. This means that the soil property distribution according to the soil layer and tillage depth [3,4,5] are the fundamental key factors that have the greatest influence on the working performance and mechanical load of agricultural tractors. Previous studies on tractor load have been mainly conducted based on the prediction and analysis of tillage force. The tillage force is the force required to pull the attached implement in the direction of tillage of the tractor and is mainly affected by the travel speed (i.e., transmission gear selection), implement geometry, tillage depth, and soil environments (e.g., soil texture, soil properties, water content). Most tillage implements manufacturers research to improve geometry to reduce tillage resistance, fuel consumption, and wear during tillage operations. The topics of energy conversion and mechanical efficiency during tillage operations are very important, as tractor manufacturers focus on designing the reliability of the power transmission caused by high tillage forces and providing adequate power to overcome tillage resistance. Therefore, it has been largely divided into implement-dependent and tractor-dependent load studies.

Various studies using classical theoretical formulas or tillage force measurement systems have been conducted to study the perspective of tillage implement-dependent loads, which means the design parameters and operating conditions of the attached implement. In some studies, soil bin experiments were conducted to evaluate the design parameters (e.g., tine spacing, rake angle, tillage width, surface treatment) and operation conditions (e.g., tillage depth, gang angle) of the tillage implement on tillage force and working performance [6,7,8,9,10,11,12,13,14]. In another study, an experimental study was conducted to develop a specific draft estimation model for offset disc harrows in a soil bin test environment [15]. In another study, a classical draft force prediction model was developed for disk plows [16].

On the other hand, vehicle-dependent load studies on the effect of tractor driving conditions such as operation type, transmission gear selection, and tillage depth on the required force and power have been mainly conducted through field tests. Basically, the study of the mechanical load through field tests performed dynamic load analysis in terms of the tractor power depending on the agricultural operation type or gear selection [17,18]. Lee [19] analyzed the PTO (power take-off) shaft load of a 75 kW agricultural tractor in rotary tillage and baler operation. Their results showed that a sharp increase in shaft torque due to PTO gear selection or an increase in the rotational speed of the PTO shaft increased the damage of the PTO shaft by 1.45 to 2.31 times. In other studies, the effect of gear selection was analyzed during deep tillage or rotary tillage operations on the mechanical load and fuel efficiency of the transmission and PTO input shaft [20,21]. It was confirmed that the mechanical load on the transmission and PTO input shaft increased by 19.47 to 22.97 times depending on the gear selection during rotary operation and that the fuel consumption during deep tillage increased by up to 2.15 times. In another study, Kim [22] analyzed the effect of gear selection and soil water contents on the mechanical load of a 78 kW agricultural tractor during moldboard plowing. The test site was divided into 3 m sizes, and the water contents were measured in all sections. Then, the mechanical load data measured by matching with the plowing section were classified and analyzed according to the water contents. As a result of field test data analysis, the lower the water content was, the higher the engine power and fuel efficiency. Research on the development of an embedded digital draft force, slip, and wheel torque indicator system was conducted to measure the wheel axle load used in the design of the tractor, as well as the tillage force required for using the tillage implement and the engine power requirement for power source selection [23,24]. In some studies, performance evaluation studies were conducted through field tests in various working environments. Vidas [25] analyzed the efficiency of disc harrow adjustment for stubble tillage quality and fuel consumption, considering exchangeable parameters such as travel speed, disc angle, and tillage depth. In another study, Balsari [26] conducted a performance evaluation of a tractor-power harrow system under different working conditions focused on PTO torque and draft force.

Although some studies have been conducted on the load according to the tractor driving conditions at the same test site [27,28], studies on how the soil environment with different characteristics in terms of soil depth distribution affects the load and performance of the tractor during tillage work have not been conducted. In addition, it is not clear to what extent the tillage depth and soil characteristics depend on the use of a specific tractor. The research reproducibility is low, making it difficult to design load. Therefore, if the effect of the working environments on the tractor performance becomes clear through this study, it will be useful information for studies related to the reliability of agricultural machinery, such as axle load prediction [29] and component life evaluation [30], and the tillage performance of the attached implement can be analyzed at the same time.

This study presents the results of an extensive experimental analysis planned to investigate the relationship between tractor performance and working environments by tillage depth during moldboard plow. This study aimed to analyze the tillage depth effect on the performance of tractor-moldboard systems under various working environments by tillage depth. The specific objectives of this study were to: (1) analyze the working environment by focusing on the soil properties based on depth distribution; (2) develop a field load measurement system and measure the load parameters during tillage operation, and; (3) analyze the tillage depth effect on the working load and performance evaluation (e.g., power transmission efficiency, traction performance, and lugging ability) of agricultural tractors.

## 2. Materials and Methods

### 2.1. Tractor-Implement System

**Figure 1:**
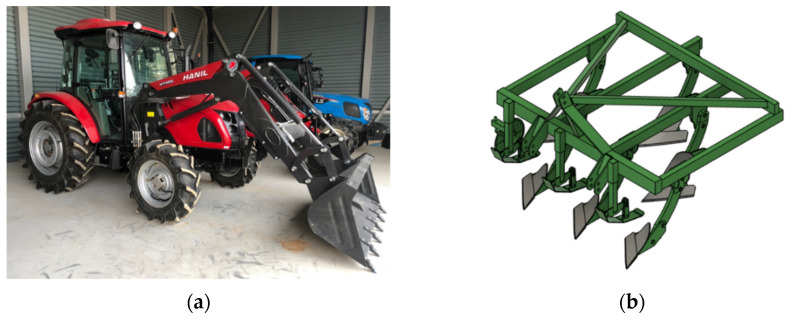
Tractor-implement system: (**a**) 42 kW class agricultural tractor (TX58, TYM); (**b**) 6-rows moldboard plow (WJSP-6S, Woongjin).

A 4WD type 42 kW class agricultural tractor (TX58, Tong Yang Moolsan, Gongju, Korea) that conforms to the OECD standard was used in the field experiments with a rated power of 35.6 kW and a maximum torque of 211.8 Nm, as shown in Figure 1a. The tractor’s empty vehicle weight was 2615 kg, but the total weight, including the attached data acquisition system (229 kg) in the cabin, a tillage force measurement system (310 kg), and a front loader (450 kg) (HIT400 L, Hanil Industry Co, Ltd., Gyeongsan, Korea), was 3894 kg. The overall dimensions of the agricultural tractor body were 3695 mm × 1848 mm × 2560 mm (length × width × height). The agricultural tractor used for the field experiment was equipped with a mechanical transmission power shuttle. In addition, the tractor can shift a total of 48 gear selections (24 forwards and 24 reverses) according to tillage operation through a combination of 4 main and 6 sub gear stages, and the maximum speed is 33.8 km/h. The specifications of the agricultural tractor are shown in Table 1. 

Figure 1b shows a 6-row moldboard plow (WJSP-6S, Woongjin Machinery, Gimje, Korea), often used in soil-machine interactions studies. Moldboard plows are a high-load operation compared to other agricultural operations; thus, they are suitable for soil–tool interaction studies of agricultural machinery. The moldboard plow has a maximum tillage depth of 20 cm, and the dimensions are 1930 × 1800 mm × 1235 mm (length × width × height). The detailed specifications of the attachment implement are shown in Table 2.

### 2.2. Field Load Measurement System

**Figure 2:**
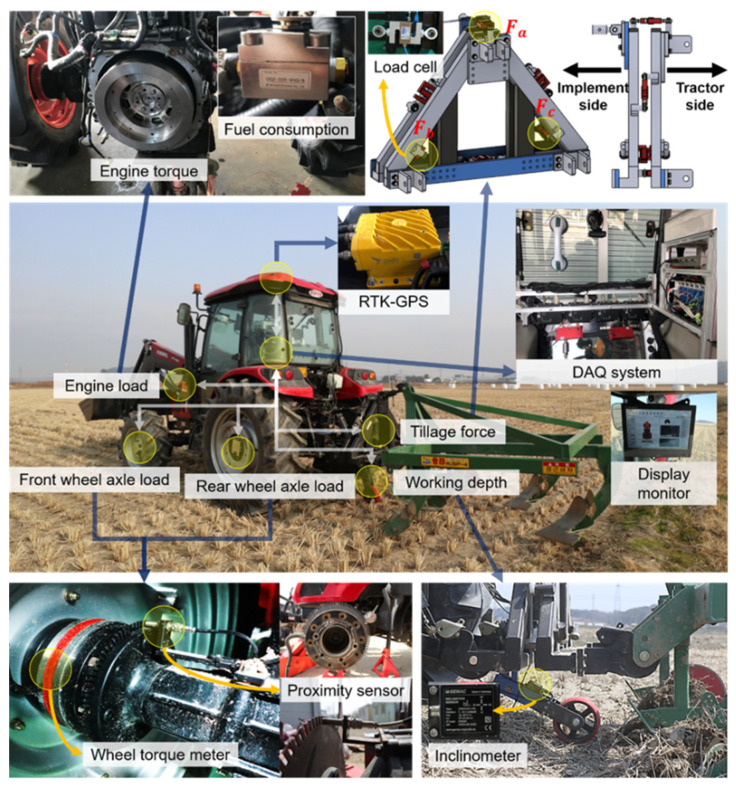
Configuration of field load measurement system.

Figure 2 shows the overall configuration of the field load measurement system developed for field experiments and the installation location of each measurement part. The data acquisition system used Dewesoft X (Dewesoft 3X, Dewesoft, Trbovlje, Slovenia) to synchronize and extract field measurement data at a sampling rate of 1000 Hz. The field load measurement system provided for the study the effect of soil working environment on the load of an agricultural tractor during moldboard plow, and consisted of an engine part (e.g., engine torque, engine speed, fuel consumption), wheel axle part (e.g., wheel axle torque, wheel axle rotational speed), a draft force measurement system, a tillage depth measurement system, and a real-time kinematic global positioning system (RTK-GPS) (Duro Inertial, Swift Navigation, San Francisco, CA, USA) for measuring travel speed of moldboard plow. The detailed specifications of the sensors constituting the field load measurement system are shown in Table 3.

Slip ratio can be obtained using the following equations.

(1) $$V_{\mathrm{th}}=\frac{{\pi D}_{\mathrm{wr}}N_{\mathrm{wr}}\mathrm{GR}3.6}{60},$$

(2) $$S=\left(\frac{V_{\mathrm{th}}-V_{a}}{V_{\mathrm{th}}}\right)100,$$

where $V_{\mathrm{th}}$ is the theoretical speed of the tractor in the working direction (km/h), $D_{\mathrm{wr}}$ is the diameter of the rear wheel axle (m), $N_{\mathrm{wr}}$ is the wheel axle rotational speed as calculated by proximity sensors (rpm), GR is the gear ratio, S is the slip ratio (%), and $V_{a}$ is the travel speed of the tractor measured by RKT-GPS (km/h).

To measure the load data more accurately compared to previous studies that performed field tests using only CAN communication, the engine load measurement part consisted of a torque transducer and flowmeter for the direct measurement of engine torque ($T_{e}$) and fuel consumption (FC). To measure engine torque, a 5 kNm strain gauge-type torque transducer was installed on the flexplate, and torque data were measured by contactless sensor telemetry through an amplifier. The engine rotation speed ($N_{e}$) was measured by wireless controller area network (CAN) communication. In addition, the lugging ability of a tractor refers to the ability of the engine to overcome a sudden large load during tillage operation without easily stopping it. This is a major performance indicator of how much load is generated and can be overcome in various working environments in the tractor-implement system [2]. Lugging ability-related parameters can be obtained as follows.

(3) $$P_{e}=\frac{2{\pi T}_{e}N_{e}}{60000},$$

where $P_{e}$ is the power requirement of the engine (kW), $T_{e}$ is the engine torque (Nm) measured by the flex plate torque transducer, and $N_{e}$ is the engine speed (rpm) measured by controller area network (CAN) communication.

(4) $$\eta =\frac{P_{e}}{P_{r}}\times 100,$$

(5) $$\delta =\frac{N_{e}}{N_{r}}\times 100,$$

(6) $$\mu =\frac{D_{n}-D_{r}}{D_{r}}\times 100,$$

where η is the engine power ratio (%), $P_{r}$ is the rated engine power (kW), δ is the engine speed ratio (%), $N_{r}$ is the rated engine speed (rpm), μ is the lugging ability (%), $D_{n}$ is the draft force at engine speed ‘n’ (kN), and $D_{r}$ is the draft force at rated engine speed (kN).

Fuel consumption (OG2-SS5-VHQ-B, Titan Enterprises, Sherborne, UK) was measured by installing an oval gear flowmeter at the engine fuel injection inlet and outlet. Specific fuel consumption (SFC), which is mainly used for performance indicators of fuel consumption, can be obtained from the following equations. The fuel cost was also calculated through a literature study [21].

(7) $$\mathrm{FC}=\frac{60\left(F_{\mathrm{in}}-F_{\mathrm{out}}\right)}{0.83},$$

(8) $$\mathrm{SFC}=\frac{\mathrm{FC}}{P_{e}},$$

(9) $$\mathrm{PR}=\frac{\mathrm{TS}\times \mathrm{WW}}{10},$$

(10) $$\mathrm{Fuel}\;\cos t=\mathrm{FC}\times \frac{1}{\mathrm{PR}}\times \mathrm{FP},$$

where FC is the fuel consumption of the engine (kg/h), $F_{\mathrm{in}}$ is the flow rate (L/min) measured by the flow meter installed on the fuel inlet of the engine, $F_{\mathrm{out}}$ is the flow rate (L/min) measured by the flow meter installed on the fuel outlet of the engine, SFC is the specific fuel consumption (g/kWh), PR is the productivity rate (ha/h), TS is the travel speed measured by RTK-GPS (km/h), WW is the working width (m), fuel cost ($/ha), and FP is the fuel price (1.23 $/L).

The wheel axle load (wheel axle torque and rotational speed) measurement part consisted of a flange-type torque transducer (PCM16, MANNER, Spaichingen, Germany) and a proximity sensor (CYGTS211B-PO2, Chen Yang Technologies GmbH & Co. KG, Finsing, Germany). A torque transducer, proximity sensor, and antenna with an integrated amplifier were installed inside and outside each wheel axle shaft to measure the wheel axle load. The wheel axle torque data from each torque transducer were amplified by internal amplifiers and transmitted wirelessly to a stationary antenna. A tooth profile of a helical gear or spur gear and a tooth thickness of 3 mm or more is required to measure the wheel axle rotational speed.

(11) $$P_{w}=\frac{2{\pi T}_{w}N_{w}}{60000},$$

where $P_{w}$ is the power requirement of the wheel axle (kW), $T_{w}$ is the wheel axle torque (Nm) measured by the wheel torque meter, and $N_{w}$ is the wheel axle rotational speed (rpm) measured by the proximity sensor.

The tillage force measurement system was configured to measure draft force by using 6 tensile compression-type universal load cells (SBA-2T, CAS, Yangju, Korea) on a triangular jig. It was located between the link for attaching the rear of the tractor and the implement [31,32]. The equation for calculating the draft force from the measured values obtained from the load cell attached to the tillage force measurement system is as follows.

(12) $$D=F_{a}+F_{b}+F_{c},$$

where D is the draft force, which is the sum of the forces applied to the system in the horizontal direction (kN), and $F_{a}$, $F_{b}$, and $F_{c}$ are the measured forces at the load cell for each attachment position depicted.

In addition, drawbar power and tractive efficiency were analyzed to evaluate traction performance, which is a major performance indicator of the tractor as follows.

(13) $$P_{\mathrm{db}}=\frac{D\times V_{a}}{3},$$

(14) $$\mathrm{TE}=\frac{P_{\mathrm{db}}}{P_{w}}\times 100,$$

where $P_{\mathrm{db}}$ is the drawbar power (kg/h) and TE is the tractive efficiency(%).

To analyze the effect of tillage depth in studies related to tractor tillage operation, the work is generally performed after fixing the hitch height at the target tillage depth. However, since the soil in agricultural fields is irregular and bumpy, a precise real-time tillage measurement system is required. The tillage depth measurement system consisted of an inclinometer (IS2MA090-U-BL, GEMACsensors, Chemnitz, Germany) for measuring the vertical penetration depth of the implement during tillage operation. The inclinometer attached to the lower link of the depth measurement system was used to measure the angle of the lower link according to the vertical penetration depth that occurs when the attached implement penetrates the soil. In the case of a change in the angle of inclination due to the occurrence of tillage depth, the measured angle of the lower link was drastically reduced compared to the initial angle $\theta_{1}$ at zero tillage depth. The tillage depth was obtained using the following equation [27]:

(15) $$d=L_{L}\sin\theta_{1}-L_{L}\sin\theta_{2},$$

where d is the tillage depth, $L_{L}$ is the length of the lower link of the tillage depth measurement system (m), $\theta_{1}$ is the initial angle of the lower link measured using the inclination sensor when the tillage depth is zero (deg), and $\theta_{2}$ is the angle of the lower link measured when the tillage depth occurs (deg).

### 2.3. Experimental Design

**Figure 3:**
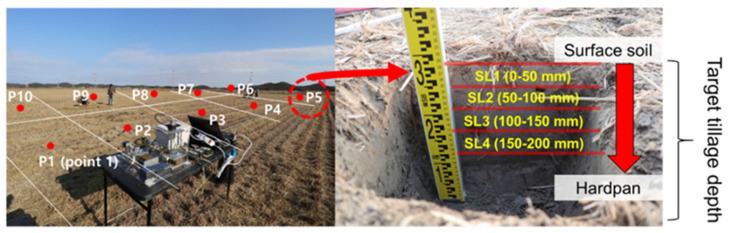
Soil environment analysis procedure considering depth distribution: Soil sampling using uniformed grid sampling method.

In the soil measurement process, soil properties were measured using the uniform grid sampling method [33]. To consider the change in soil properties distributed according to the target tillage depth, each test site was divided into a uniform grid with 5–10 measurement points. In addition, 1 measurement point was divided into 4 soil layers of a 50 mm deep layer (SL1: 0–50 mm, SL2: 50–100 mm, SL3: 100–150 mm, and SL4: 150–200 mm), as shown in Figure 3. Soil properties measured through field measurements were bulk density (γ), water content (WC), porosity (ƞ), cone index (CI), shear strength ($\tau_{f}$), and Atterberg limits (i.e., plastic index, plastic limits, liquid limits).

First, the soil sampling was performed using a 100 mL stainless steel tube (51 mm in diameter and 50 mm in height) (DIK-1801, Daiki Rika Kogyo Co., Ltd., Akagidai, Japan) and a soil sampling apparatus (DIK-1815, Daiki Rika Kogyo Co., Ltd., Akagidai, Japan). Bulk density was obtained by measuring the weight of 100 mL of sampled soil with an electronic scale (EK-610i, AND, Seoul, Korea). In addition, soil samples weighed for bulk density measurement were then measured for porosity with volume fraction using an actual volumenometer (DIK-1150, Daiki Rika Kogyo Co., Ltd., Akagidai, Japan). Afterward, the sampled soil specimen was dried at a temperature of 110 °C for 24 h using the oven drying method for measuring the water content [34] using an experimental oven (SH-DO-100FGB, Samheung Energy, Sejong, Korea). The dried soil was analyzed for soil texture (i.e., particle size distribution) by the USDA method [35] through sieve analysis using a sieve shaker (HJ-4560, Heungjin, Gimpo, Korea) [36]. To determine the plastic index, which indicated the level of compression and stickiness in the soil environment [37], the Atterberg limits test was performed to obtain the plastic index using an electric liquid-limit tester (HJ-4105, Heungjin, Gimpo, Korea) [38]. To measure the cone index and shear strength, a cone penetration test and shear vane test were performed using a cone penetrometer (DIK-5532, Daiki Rika Kogyo Co., Ltd., Akagidai, Japan) [39,40] and shear ring-type soil resistance meter (DIK-5503, Daiki Rika Kogyo Co., Ltd., Saitama, Japan).

To analyze the effect of the soil working environment on the load and working performance of agricultural tractors, field experiments were conducted in 3 different agricultural field environments (First test site: 36°22′01.7″ N and 127°21′18.6″ E, second test site: 37°00′42.7″ N and 126°21′18.5″ E, third test site: 37°56′11.3″ N and 127°46′58.2″ E). Before performing tillage using a measuring tractor equipped with a field load measurement system, soil property measurement tests were performed. Moreover, field experiments were performed based on the 3× 3 split-plot design under 3 test conditions and 3 tillage depths (12, 16, and 20 cm). Since the field load measurement system used in this study can measure the tillage depth in real-time, it does not prevent the vertical movement of the 3-point hitch from performing it at the target tillage depth reported in prior related studies. Instead, the 3-point hitch was controlled to perform the tillage operation at a target tillage depth range of 12–20 cm. To analyze the effect of tillage depth and soil properties, the results of the field measurement data were arranged in ascending order according to tillage depth. The measured field test data irregularities were removed using the group frequency distribution method to clearly analyze the influence of soil properties according to 3 tillage depth levels on load-related parameters. During moldboard plowing, the agricultural tractor selected an M3 gear with a theoretical speed of 7.9 km/h on a straight working path and operated in 4-wheel-drive mode under full-throttle, not at rated engine speed.

In addition, to analyze the tractor load under the no-load condition as a basic comparison group, the driving load in the same gear selection was additionally measured at the paddy field. Performance evaluation of tractor-tillage implement systems was generally performed through analysis of power transmission efficiency (PTE), tractive efficiency (TE), and lugging ability with torque rise [2]. Therefore, the performance evaluation of the tractor-implement system was performed by analyzing the load data measured in various working environments and tillage depths.

## 3. Results and Discussion

### 3.1. Soil Collection Data Analysis

The specific overall measurement results of the soil properties according to the soil layer are shown in Table 4, Table 5 and Table 6. As a result of soil texture analysis for each field, the first test site was analyzed with sandy loam (sand 68%, silt 20%, clay 12%), the second test site was analyzed with loam (40% sand, 48% silt, 12% clay), and the third test site was analyzed with clay loam (40% sand, 28% silt, 32% clay). Bulk density showed 1544–1800 $\mathrm{kg}/m^{3}$, 1519–1898 $\mathrm{kg}/m^{3}$, and 1899–2021 $\mathrm{kg}/m^{3}$ analysis results in sandy loam, loam, and clay loam fields, respectively. The bulk density results were generally higher than the range of 1200–1500 $\mathrm{kg}/m^{3}$, which was the bulk density value reported in previous studies performed on soil bins [41,42,43] and showed similar values to the range of 1500–2100 $\mathrm{kg}/m^{3}$ reported in studies using actual field experiments [27,44]. Porosity was 0.47–0.53, 0.41–0.54, and 0.34–0.39 in sandy loam, loam, clay loam fields, respectively, and it was confirmed that as the soil layer deepened, it decreased up to 24.1% compared to the topsoil. In addition, the water content showed values of 15.5–19.4%, 24.5–32.2%, and 20.2–26.5% in sandy loam, loam, and clay loam fields, respectively. In general, the deeper the soil layer was, the lower the water content compared to topsoil due to high soil compaction and low porosity. Loam field has dry conditions with high porosity, and the moisture content increases as the soil layer deepens, showing an opposite trend to loam and clay loam test sites. For the Atterberg limits test for relative evaluation of compressible and stickiness characteristics of test sites, the plastic index results were 6.89 (15.19% plastic limits, 22.08% liquid limits), 13.49 (21.1% plastic limits, 34.59% liquid limits), and 13.95 (18.99% plastic limits, 32.94% liquid limits) in sandy loam, loam, and clay loam fields, respectively. Sandy loam field was analyzed as having partly cohesive characteristics, and loam and clay loam fields were analyzed as having relatively high cohesive characteristics [37].

The cone index results showed ranges of 560.4–897.3 kPa, 471.2–1798.1 kPa, and 680.8–1915.5 kPa in sandy loam, loam, and clay loam, respectively. Although the average cone index value was different for each field, it was confirmed that a hardpan was formed at a depth of SL3–SL4 (10–20 cm) in all test sites. In particular, in the case of loam and clay loam, it was confirmed that the average cone index in SL3–SL4 compared to SL1–SL2 corresponding to the plow layer increased rapidly by 2.8–3.8 times. Additionally, the shear strength showed ranges of 23.8–41.4 $\mathrm{kN}/m^{2}$, 23.9–78.4 $\mathrm{kN}/m^{2}$, and 64.8–87.7 $\mathrm{kN}/m^{2}$ in sandy loam, loam, and clay loam, respectively.

### 3.2. Driving Load Analysis

**Figure 4:**
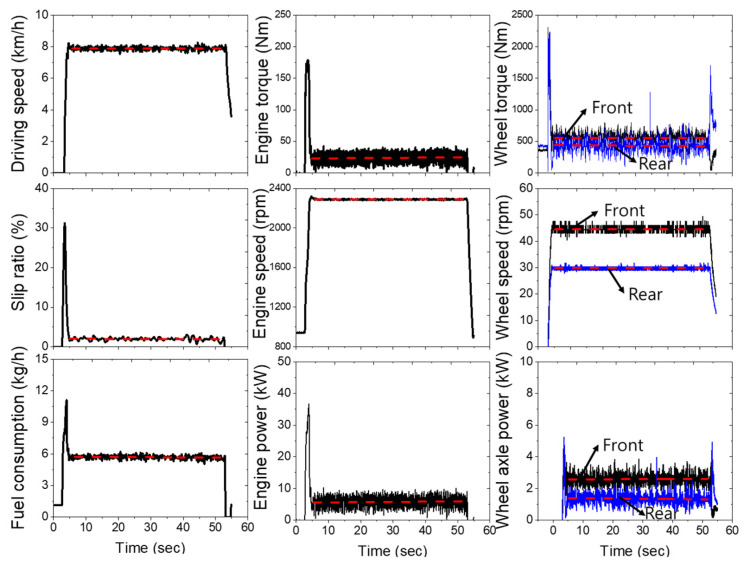
Driving load of 42 kW agricultural tractor at M3 gear selection.

Figure 4 shows the measurement result of the driving load under no-load conditions. During straight path driving, the average driving speed under the no-load condition was 7.87 km/h with a slip ratio of 2%. In the case of the engine load, the average engine torque of the tractor was 22.8 Nm, the average engine speed was 2290 rpm, and approximately 5.5 kW of engine power was used (15.4% engine power ratio), with an average fuel consumption of 5.73 kg/h. In the case of the wheel axle load, the power requirement for the front axle was 2.6 kW (wheel axle torque of 553.1 Nm, wheel axle speed of 44.5 rpm), and the power requirement for the rear axle was 1.4 kW (wheel axle torque of 444.4 Nm, wheel axle speed of 29.8 rpm). The front wheel axle load tends to be higher than the rear wheel axle load because a weighed ballast or a front loader is attached to the front of the tractor to prevent problems such as overturning due to the effect of the pitch angle by load transfer.

### 3.3. Working Load Analysis

#### 3.3.1. Tillage Depth and Travel Speed

**Figure 5:**
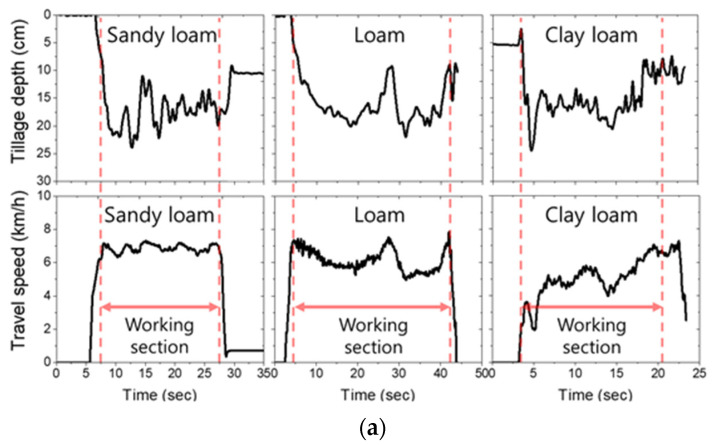

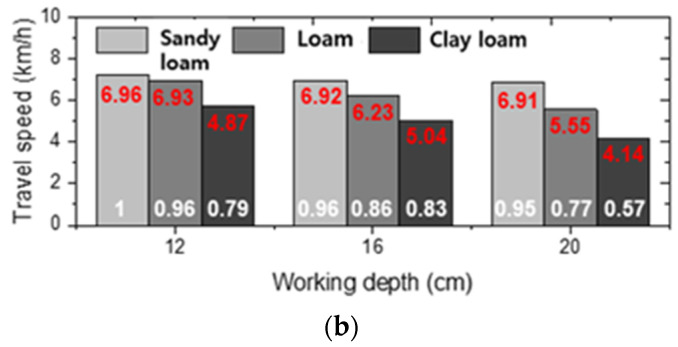
(**a**) Field measurements results of tillage depth and travel speed under various field sites; (**b**) Comparison analysis of travel speed by tillage depth and test sites.

The tillage depth and travel speed results during moldboard plowing are shown in Figure 5a. The overall mean tillage depths of each test field were 17.5 ± 2.8 cm in sandy loam, 16.3 ± 3.1 cm in loam, and 15.2 ± 3.6 cm in clay loam. As a result of the measurement data analysis, it was confirmed that the tillage depth was properly carried out in the hardpan range based on cone penetration test results (12–20 cm). In the case of the travel speed, the overall mean travel speed of each test field was 6.9 ± 0.1 km/h (6.8–7.1 km/h) in sandy loam, 6.2 ± 0.5 km/h (5.5–6.9 km/h) in loam, and 4.8 ± 0.4 km/h (4.1–5.6 km/h) in clay loam. Based on these results, it was confirmed that the travel speed decreased in the range of 12.7–39.2% from the theoretical speed of 7.9 km/h in M3 gear selection due to the influence of soil properties at a tillage depth of 20 cm. It was confirmed that the travel speed result at a tillage depth of 20 cm of clay loam was reduced by 0.57 times compared to that of sandy loam at a tillage depth of 12 cm, as shown in Figure 5b.

#### 3.3.2. Draft Force

**Figure 6:**
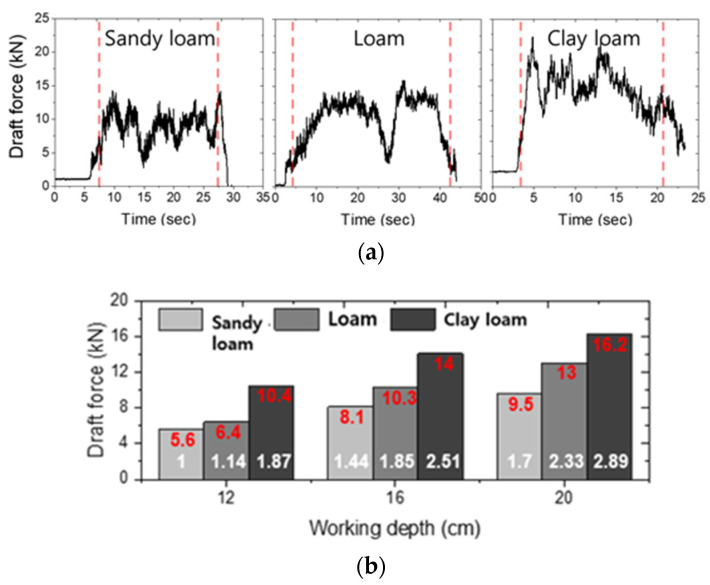
(**a**) Field measurements results of draft force under various field sites; (**b**) Comparison analysis of draft force by tillage depth and test sites.

The results of draft force according to tillage depth and test sites are shown in Figure 6a. The overall mean draft force in each test field was 8.1 ± 1.4 kN (5.6–9.5 kN) in sandy loam, 10.1 ± 2.5 kN (6.4–13 kN) in loam and 13.9 ± 2.5 kN (10.4–17.7 kN) in clay loam. Compared to sandy loam, loam and clay loam showed 1.24 times and 1.71 times higher overall mean draft forces in the hardpan section. This confirms that various working environments were highly affected by the draft force according to tillage depth. As shown in Figure 6b, it was confirmed that the result at a tillage depth of 20 cm of clay loam increased rapidly by 2.89 times compared to that of sandy loam at a tillage depth of 12 cm.

#### 3.3.3. Engine Load with Fuel Consumption

**Figure 7:**
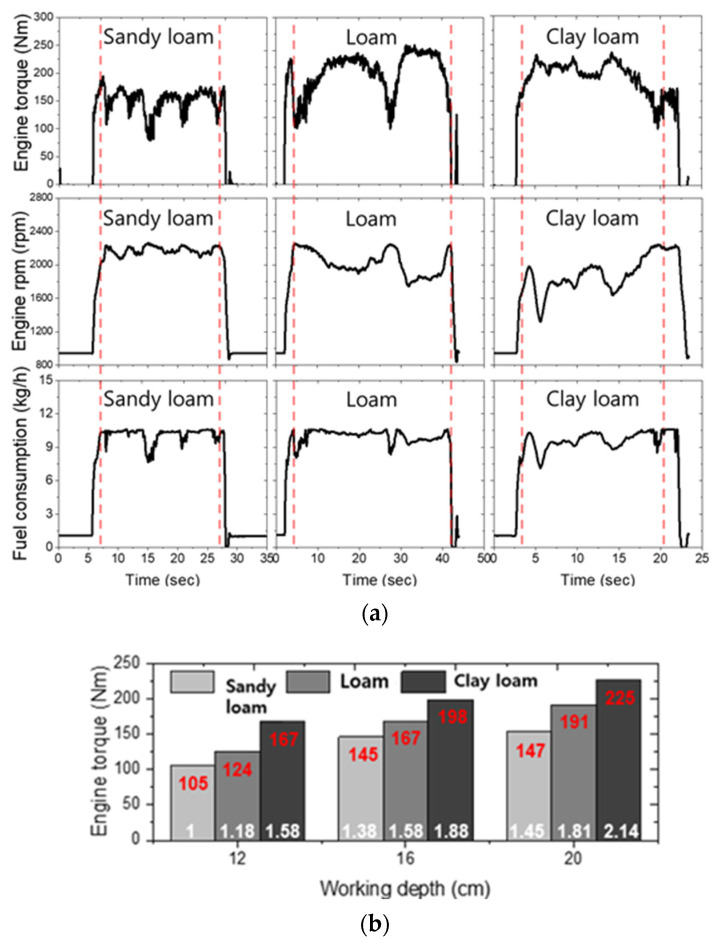
(**a**) Field measurements results of the engine load with fuel consumption under various field sites; (**b**) Comparison analysis of engine torque by tillage depth and test sites.

The engine load and fuel consumption results are shown in Figure 7a. The overall mean engine torque in the tillage depth section of 12–20 cm was 139.5 ± 18.4 Nm (105.5–155.3 Nm), 165.1 ± 24.3 Nm (124.9–192.5 Nm), and 201.5 ± 16.3 Nm (167.4–225.9 Nm) in sandy loam, loam, and clay loam, respectively. As shown in Figure 7b, the engine torque increased up to 2.14 times compared to that of sandy loam at a tillage depth of 12 cm. The engine torque increased up to 2.14 times compared to that of sandy loam at a tillage depth of 12 cm. The engine speed showed a tendency to decrease rapidly as the tillage depth increased. The smallest reduction in engine speed was from 2236.2 rpm to 2176 rpm in sandy loam (up to a 1.1% reduction compared to the rated engine speed). In the case of loam, the engine speed decreased from 2213.9 rpm to 1902.7 rpm (up to 13.6% reduction compared to the rated engine speed). The maximum engine speed decreased from 2024.9 rpm to 1668.3 rpm in clay loam (up to 25.4% reduction compared to the rated engine speed).

In general, the literature related to fuel consumption in agricultural machinery mainly performed a comparative analysis of fuel consumption according to the load ratio obtained from CAN data or tillage force generated under different driving test design conditions in the same test field [21,45,46]. In this study, fuel consumption analysis was performed considering the tractor’s working load, such as engine torque and wheel axle torque, and tillage force and tillage depth measured in real-time in various field environments. The overall mean fuel consumption was 9.8 ± 0.6 kg/h (8.5–10.2 kg/h), 10.1 ± 0.2 kg/h (9.8–10.3 kg/h), and 9.5 ± 0.3 kg/h (8.9–9.7 kg/h) in sandy loam, loam, and clay loam, respectively. In loam and clay loam fields, as the depth of the tillage increases, the fuel consumption decreases, but in the case of sandy loam, the average fuel consumption increases. This is lower than the rated engine speed of loam and clay loam, and it is judged to be affected by engine speed higher than the rated engine speed in sandy loam, which is a relatively soft soil environment.

#### 3.3.4. Wheel Axle Load with Slip Ratio

**Figure 8:**
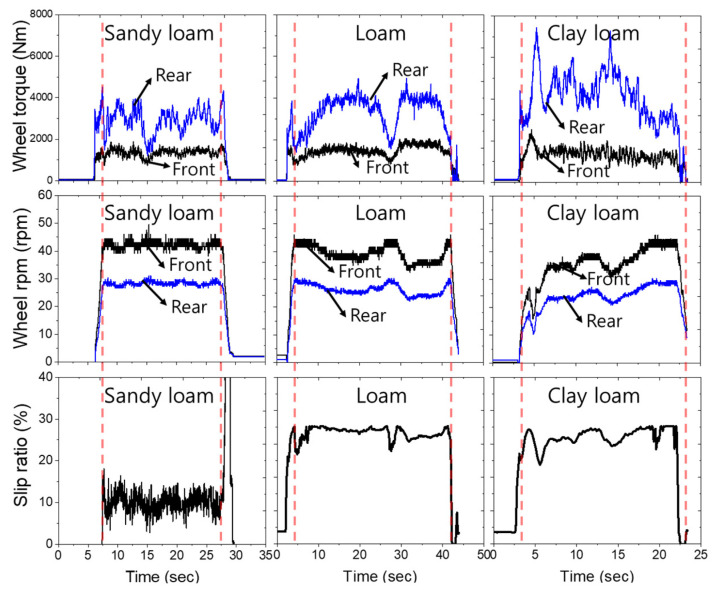
Field measurements results of wheel axle load and slip ratio under various field sites.

The wheel axle load and slip ratio results are shown in Figure 8. The overall mean front wheel axle torque in the tillage depth section of 12–20 cm was 1297.7 ± 129.3 Nm (1085.6–1448.4 Nm), 1461.9 ± 164.5 Nm (1224.4–1662.3 Nm), and 1373.1 ± 61.1 Nm (1246.3–1453.2 Nm) in sandy loam, loam, and clay loam, respectively. In the case of the front-wheel axle torque, the average torques of loam and clay loam were almost similar. The overall mean front wheel speeds in the tillage depth section of 12–20 cm were 42.6 ± 0.4 rpm (42.1–43.5 rpm), 39.8 ± 2.4 rpm (35.8–43 rpm), and 34.6 ± 1.9 rpm (31.3–38.1 rpm) in sandy loam, loam, and clay loam, respectively. The wheel axle rotational speed was affected by the decrease in engine speed, so when the tillage depth increased from 12 to 20 cm, it decreased up to 4.3%, 10.4%, and 22.3% at sandy loam, loam, and clay loam, respectively, compared to the wheel speed under driving load conditions.

**Figure 9:**
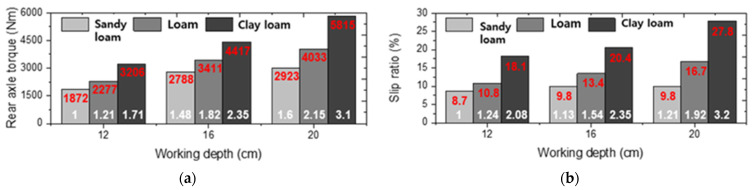
Comparison analysis by tillage depth and test sites: (**a**) rear wheel axle torque; (**b**) slip ratio.

In the case of the rear wheel axle load, the overall mean rear wheel axle torque in the tillage depth section of 12–20 cm was 2649 ± 435 Nm (1872.2–3075.4 Nm), 3256.2 ± 681.5 Nm (2277.3–4033 Nm), and 4442.8 ± 875.4 Nm (3206.1–5815.9 Nm) at sandy loam, loam, and clay loam, respectively. Although most engine torque is generated within the maximum torque point on the engine performance curve, the rear axle torque tends to increase rapidly without limitation. In particular, loam and clay loam were 1.22, 1.67 times higher than the overall average rear wheel axle torque of sandy loam. As seen from Figure 9a, the rear wheel axle torque, which is most importantly utilized as the tractor design load, increased by an average of 3.1 times in clay loam compared to sandy loam, depending on the field environment and tillage depth. The overall mean rear wheel speeds in the tillage depth section of 12–20 cm were 28.5 ± 0.2 rpm (28.3–29.3 rpm), 26.7 ± 1.6 rpm (24.7–28.8 rpm), and 23.1 ± 1.3 rpm (21.1–25.5 rpm) in sandy loam, loam, and clay loam, respectively.

In addition, the overall mean slip ratios of each test field were 9.5 ± 0.5% (8.5–10%) in sandy loam, 13.4 ± 2.4% (10.1–16.7%) in loam, and 22.3 ± 3.8% (18.1–28%) in clay loam. The overall average slip ratios of loam and clay loam were 1.41 times and 2.34 times higher than those of sandy loam, respectively. As shown in Figure 9b, the mean slip ratio of up to 1.92–3.2 times higher occurred in loam and clay loam compared to that of sandy loam at a tillage depth of 12 cm, which had the lowest average slip ratio. Normally, traction of off-road machinery is defined as the ability of the vehicle’s wheel to develop efficient soil thrust force to overcome resistance force (e.g., draft force) [47,48]. In other words, the higher the soil resistance, the lower the traction force and the higher the slip ratio. From the test results, it is judged that a higher draft force occurred in clay loam, resulting in a higher slip ratio than in sandy loam and loam.

### 3.4. Performance Evaluation of Agricultural Tractor

#### 3.4.1. Traction Performance

**Figure 10:**
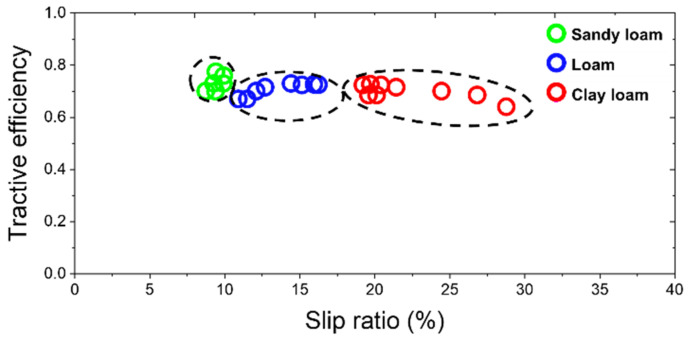
Tractive efficiency by slip ratio and test sites.

Depending on the field environment conditions, the traction performance was basically affected by the average 2.3 times increase in the slip ratio occurrence range. As shown in Figure 10, the overall mean tractive efficiency in the tillage depth section of 12–20 cm was 0.7 ± 0.02 (0.65–0.73), 0.68 ± 0.04 (0.61–0.74), and 0.61 ± 0.03 (0.56–0.66) in sandy loam, loam, and clay loam, respectively. As soil hardness and compaction increased over sandy loam to clay loam, it was confirmed that the average tractive efficiency decreased by an average of 12.9% and a maximum of 23.3%. Compared with the results of previous studies of slip-based traction performance curves [49], it was confirmed that the maximum tractive efficiency occurred at a slip ratio of 10%, and the overall data trend was that the tractive efficiency decreased as the slip ratio increased.

#### 3.4.2. Lugging Ability with Torque Rise

**Figure 11:**
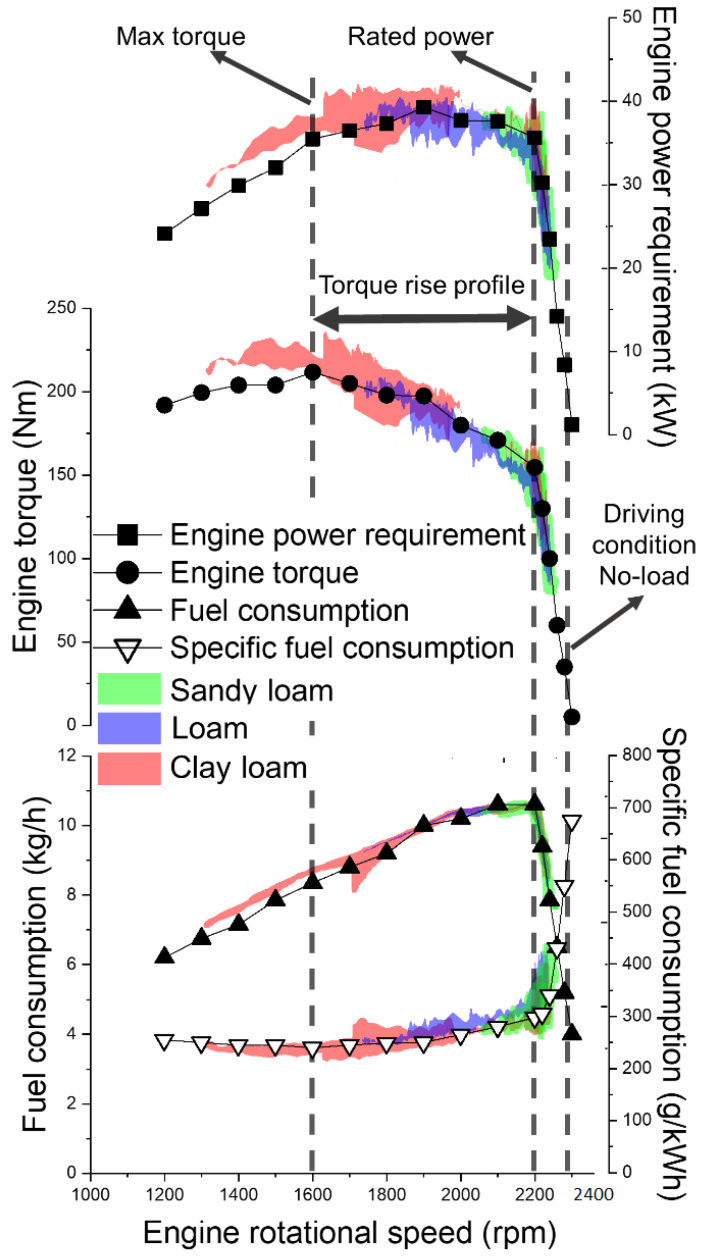
Comparative analysis of measurement data on tractor engine performance curve.

Figure 11 shows the distribution of the measured working load parameters according to engine rpm on the engine performance curve calibrated by KOAT (Korea Agriculture Technology Promotion Agency) in various working environments and target tillage depths (12–20 cm).

**Figure 12:**
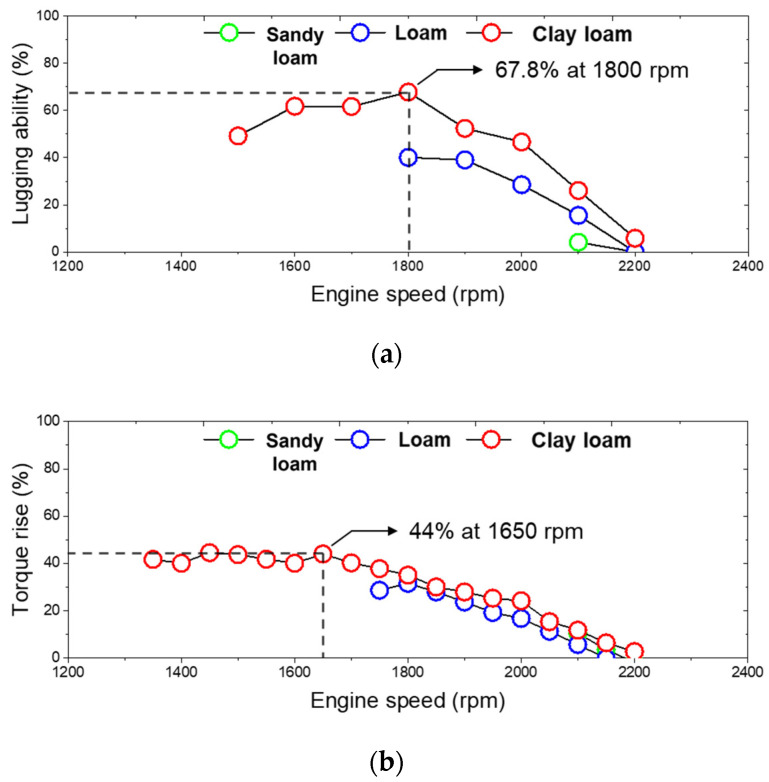
(**a**) Lugging ability; (**b**) torque rise ranges.

The maximum torque of the tractor engine used in this study was 211.8 Nm at 1600 rpm. The engine torque was measured in the ranges of 50–73.6%, 59.1–91.2%, and 50–107% compared to the maximum engine torque at sandy loam, loam, and clay loam, respectively. Therefore, when a maximum draft force of 17.7 kN occurs under certain soil property conditions at the clay loam using a 6-row moldboard plow, it was determined that there was a 67.8% lugging ability that enables continuous operation even when the engine torque increase rate was increased to a maximum of 44% compared to the engine rated torque of 140 Nm at 2200 rpm, as shown in Figure 12.

#### 3.4.3. Power Transmission Efficiency

The power transmission efficiency of the 42 kW agricultural tractor according to tillage depth and test sites is shown in Table 7. The overall mean engine power requirements were 24.6–34.8 kW, 28.9–38 kW, and 34.9–39.4 kW, ranging from 58.8–82.8%, 68.8–90.4%, and 83–93.8%, respectively, compared to the engine maximum capacity of 42 kW. Except for the 12 cm shallow tillage depth section of sandy loam, which is soft and weak, it was confirmed that more than 80% of the maximum engine power was used in most sections, including the hardpan layer (16–20 cm). In the case of the rear wheel axle, the overall mean power requirements were 11.4–17.8 kW, 13.7–20.9 kW, and 17–25.6 kW, which ranged from 46.3 to 51.1%, 47.4 to 55%, and 48.7 to 64.9, respectively, compared to the engine power requirements. In the case of the front wheel axle, the overall mean power requirements were 9.8–12.8 kW, 11–12.7 kW, and 8.7–10.2 kW, which ranged from 35.7 to 39.8%, 32.9 to 38%, and 22 to 28, respectively, compared to the engine power requirements. Overall, the power required for the front axle was in a similar range, and the lowest front axle power transmission efficiency was shown in the clay loam condition. It was found that it was affected by the rapid load transfer to the rear axle by the high draft force in clay loam. The average engine power requirement in the 16–20 cm tillage depth section in sandy loam and clay loam showed only a difference of 11.6%, but it was confirmed that the required power of the rear wheel axle was up to 30.5%. This means that it is difficult to predict the exact wheel axle load and required power only by analyzing the engine load. There is little difference in different field environments in the front wheel axle, but in the case of the rear wheel axle, the axle torque and slip must be considered according to the tillage depth. In particular, when the plasticity index is high, such as in loam and clay loam, the cohesive force is very high. The tillage force is significantly different depending on the tillage depth when the hardpan is clearly formed [50]. Therefore, analysis of soil characteristics according to soil depth is essential.

The fuel efficiency of the 42 kW agricultural tractor according to tillage depth and test sites is shown in Table 8. In the engine performance curve, when the engine speed is lower than the maximum power point, the power requirement decreases, but the power requirement tends to gradually increase due to the occurrence of over torque due to the high slip ratio. Eventually, a high-power requirement occurs under fuel consumption conditions at low engine speeds, resulting in very low specific fuel consumption. In addition, in the hardest and most viscous soil environment, in clay loam, the productivity rate decreased by up to 42.7% compared to sandy loam, depending on the working depth, and it was determined that 1.81 times higher fuel cost was required. In general, it has been reported that in order to obtain maximum fuel efficiency during tillage operation, it is required that the work be performed under the conditions of using the high torque ranges in the appropriate slip range [51]. Therefore, it was analyzed that the conditions of the 8–16% slip ratio range [52] and the highest engine torque range reported as the appropriate slip ratio of the cultivated field were satisfied when the tillage was performed with a tillage depth of 16–20 cm in the loam. In the case of sandy loam, the slip ratio was ideal at about 10%, but the soil resistance was very low, resulting in little engine load. On the contrary, in the case of clay loam, a large engine load occurred, but an excessive slip ratio of 16% or more occurred.

## 4. Conclusions

This study confirmed that even if tillage operation was performed at the same tillage depth, it affected a various range of tractor working loads and working performances according to different working environments. The major findings are as follows.

1. The bulk density, cone index, and shear strength were increased by 1.25 times, 1.44–2.17 times, and 1.62–2.72 times, respectively, according to the field environment in the same soil layer. In addition, porosity decreased up to 0.73 times according to the field environment in the same soil layer. In particular, in the case of loam and clay loam, where the hardpan layer was distributed, it showed a tendency to change rapidly from a soil depth of approximately 12–13 cm. Therefore, the soil physical properties according to the target tillage depth should be considered the top priority in the performance evaluation process or field monitoring of agricultural machinery.
2. As a result of the field test, the travel speed decreased by 12.7–39.2%, and the slip ratio increased by up to 3.2 times according to tillage depth. Overall engine torque was up to 2.14 times higher in average engine torque in hard and cohesive soil environments compared to soft soil environments, and engine rotational speed was reduced by up to 0.74 times. In addition, the average rear wheel axle torque is up to 1.67 times higher.
3. From the tractor’s point of view, even when plowing at the same tillage depth, clay showed the highest draft force, and it was confirmed that the tractor could withstand the maximum load, but excessive slip of more than 16% occurred. In addition, results showed that sandy loam and loam had relatively lower soil resistance than clay loam and had a margin of use torque increase, making it possible to plow even under higher gear stage conditions. For fuel efficiency, the tractor-implemented system in this study was analyzed to use a relatively high torque range at a moderate slip of 8–16% when plowing at a tillage depth of 16–20 cm in loam.
4. From the viewpoint of the tillage implement, if the target is a soil environment such as sandy loam or loam, it is judged that the working efficiency can be further increased by additionally increasing the tillage width or changing the geometry design thus that it can be operated at a deeper tillage depth. Alternatively, it is possible to comprehensively check whether the maximum soil resistance occurs, which can serve as a basis for determining appropriate power machine matching.

The tractor-implement system performance evaluation method can be widely utilized to select an appropriate engine capacity and attached implement for agricultural tractors, optimal design, and gear selection of the power transmission system, decision support of the control system, and three-point hitch elevation control for soil alleviation of soil compaction, and so on. In addition, from the viewpoint of the attached implement, it will be possible to use it for research that can minimize the tillage force through geometry design. Based on this approach and utilization of the performance evaluation method, the optimal tractor-implement system matching and optimal design will be possible by predicting the working load according to the target soil environment.

## Figures and Tables

**Table 1:** Specification of the agricultural tractor.

Item		Specification
Weight (kg)	Empty	2615
	Total	4072
Wheel base (mm)		2155
Length (mm) × width (mm) × height (mm)		3695 × 1848 × 2560
Engine	Rated Power (kW)	35.6 @ 2000 rpm
	Maximum torque (Nm)	211.8 @ 1600 rpm
Transmission	Main	4 stages (1, 2, 3, 4)
	Sub	6 stages (C-L, C-M, C-H, L, M, H)
Tire	Size (front/rear)	11.2–20/14.9–30
	Type	Bias
Gear ratio (front wheel axle/rear wheel axle)		51:1/76:1
Maximum travel speed (km/h)		33.8

**Table 2:** Specification of the tillage implement.

Item	Specification
Product name	WJSP-6S
Manufacturing company	Woongjin
Type	Moldboard plow
Weight (kg)	370
Length (mm) × width (mm) × height (mm)	1930 × 1800 × 1235
Required power (kW)	40–52
Maximum tillage depth ranges (mm)	Up to 200
Rake angle (deg)	30.7
Single blade width (mm)	270
Share length (mm)	360
Share type	Plain coulter with spring
Number of furrows	3

**Table 3:** Specification of the sensors constituting the field load measurement system.

Item		Specification
Torque meter	Name (product/company)	PCM16/MANNER
	Measuring torque range	15 kNm
	Sampling rate	1 kHz
Proximity sensor	Name (product/company)	CYGTS211B-PO2/GmbH and Co. KG
	Rated sensing distance	≤3 mm
	Sampling rate	1 Hz to 20 kHz
	Measuring rpm range	1–20,000 rpm (using 60P/R gear)
	Operating temperature ranges	−40 to 125 °C
Load cell	Name (product/company)	SBA-2T/CAS
	Measuring capacity	2tf
	Rated Output	3.0 mV/V
	Operating temperature ranges	−30 to 80 °C
	Protection grade	IP 66
Flow meter	Name (product/company)	OG2-SS5-VHQ-B/Titan Enterprises
	Measuring capacity	4 L/min
	Operating type	Oval gear type
Inclinometer	Name (product/company)	IS2MA090-U-BI/GEMAC sensors
	Protection grade	IP 67
	Accuracy	±0.1°
	Operating temperature ranges	−40 to 80 °C
RTK GPS	Name (product/company)	Duro Inertial/Swift Navigation
	RTK accuracy: Horizontal	0.01 m + 1 ppm
	RTK accuracy: Vertical	0.015 m + 1 ppm
	Sampling rate (GPS/IMU)	10/25–200
	Network Protocol Supported	NTRIP Client

**Table 4:** Results of measurement of soil properties according to soil layers in sandy loam (first site).

Parameters	SL1	SL2	SL3	SL4
WC ^1^ (%)	15.52 ± 0.3	18.07 ± 1.01	19.6 ± 1.62	19.45 ± 0.92
η ^2^	0.53 ± 0.02	0.51 ± 0.01	0.49 ± 0.01	0.47 ± 0.03
γ ^3^$(\mathrm{kg}/m^{3}$)	1544 ± 74.4	1644 ± 36.8	1688 ± 44.4	1800 ± 114.2
CI ^4^ (kPa)	560.45 ± 130.6	725.33 ± 114.9	810.29 ± 89.5	879.3 ± 82.7
$\tau_{f\;}$^5^ (kPa)	23.8 ± 7.6	28.75 ± 3.5	36.07 ± 3.6	41.43 ± 1.8

^1^ WC: water content; ^2^ η: porosity, ^3^ γ: bulk density, ^4^ CI: cone index, ^5^ $\tau_{f}$: shear strength.

**Table 5:** Results of measurement of soil properties according to soil layers in loam (second site).

Parameters	SL1	SL2	SL3	SL4
WC ^1^ (%)	32.2 ± 0.07	34.1 ± 1.62	28.4 ± 4.5	24.5 ± 3.9
η ^2^	0.54 ± 0.01	0.52 ± 0.03	0.49 ± 0.07	0.41 ± 0.02
γ${\;}^{3}\;(\mathrm{kg}/m^{3}$)	1519 ± 75.2	1614 ± 97.4	1662 ± 179.4	1898 ± 108.1
CI ^4^ (kPa)	471.2 ± 53.8	563.14 ± 71.1	655.3 ± 109.2	1798.1 ± 491.8
$\tau_{f}$ ^5^ (kPa)	23.9 ± 4.3	30.87 ± 3.5	32.9 ± 5.2	78.4 ± 6.4

^1^ WC: water content; ^2^ η: porosity, ^3^ γ: bulk density, ^4^ CI: cone index, ^5^ $\tau_{f}$: shear strength.

**Table 6:** Results of measurement of soil properties according to soil layers in clay loam (third site).

Parameters	SL1	SL2	SL3	SL4
WC ^1^ (%)	26.56 ± 1.61	23.8 ± 1.77	22.49 ± 0.93	20.24 ± 2.21
η ^2^	0.39 ± 0.02	0.38 ± 0.02	0.36 ± 0.03	0.34 ± 0.02
γ${\;}^{3}\;(\mathrm{kg}/m^{3}$)	1899 ± 27.86	1905 ± 49.71	1955 ± 83.31	2021 ± 72.97
CI ^4^ (kPa)	680.8 ± 117.9	900.6 ± 89	1012.6 ± 143.2	1915.5 ± 258.9
$\tau_{f}$ ^5^ (kPa)	64.83 ± 7.9	63.36 ± 8.2	58.49 ± 6.7	87.74 ± 5.8

^1^ WC: water content; ^2^ η: porosity, ^3^ γ: bulk density, ^4^ CI: cone index, ^5^ $\tau_{f}$: shear strength.

**Table 7:** Results of power transmission efficiency by tillage depth and test sites.

Test Site	TD ^1^ (cm)	$P_{e}{\;}^{2}\;(\mathrm{kW})$	$PTE_{e}{\;}^{3}\;(\%)$	$P_{fw}{\;}^{4}\;(\mathrm{kW})$	$PTE_{fw\;}{\;}^{5}\;(\%)$	$P_{rw}{\;}^{6}\;(\mathrm{kW})$	$PTE_{rw}{\;}^{7}\;(\%)$
Sandy loam	12	24.6	58.8	9.8	39.8	11.4	46.3
	16	33.3	79.2	11.9	35.7	16.6	49.8
	20	34.8	82.8	12.8	36.7	17.8	51.1
Loam	12	28.9	68.8	11	38	13.7	47.4
	16	35.8	85.2	11.8	32.9	19	53
	20	38	90.4	12.7	33.4	20.9	55
Clay loam	12	34.9	83	9.8	28	17	48.7
	16	38.7	92.1	10.2	26.3	21.8	56.3
	20	39.4	93.8	8.7	22	25.6	64.9

^1^ TD: tillage depth; ^2^ $P_{e}$: engine power, ^3^ ${\mathrm{PTE}}_{e}$: power transmission efficiency of the engine compared to engine maximum power, ^4^ $P_{\mathrm{fw}}$: front wheel axle power, ^5^ ${\mathrm{PTE}}_{\mathrm{fw}}$: power transmission efficiency of front wheel axle compared to engine power, ^6^ $P_{\mathrm{rw}}$: rear wheel axle power, ^7^ ${\mathrm{PTE}}_{\mathrm{rw}}$: power transmission efficiency of rear wheel axle compared to engine power.

**Table 8:** Results of fuel efficiency by tillage depth and test sites.

Test Site	TD (cm)	FC ^1^ (kg/h)	SFC ^2^ (g/kWh)	PR ^3^ (ha/h)	Fuel Cost ($/ha)
Sandy loam	12	8.5	349.7	1.29	8.1
	16	10.1	306.2	1.24	9.9
	20	10.2	305.7	1.24	10.1
Loam	12	9.8	345.7	1.24	9.7
	16	10.3	289.7	1.12	11.3
	20	9.9	261.2	1	12.2
Clay loam	12	9.7	284.7	1.02	11.7
	16	9.7	252.1	0.9	13.2
	20	8.9	226.2	0.74	14.7

^1^ FC: fuel consumption; ^2^ SFC: specific fuel consumption, ^3^ PR: productivity rate.
